# Renin–Angiotensin–Aldosterone System: Friend or Foe—The Matter of Balance. Insight on History, Therapeutic Implications and COVID-19 Interactions

**DOI:** 10.3390/ijms22063217

**Published:** 2021-03-22

**Authors:** Fedor Simko, Jaroslav Hrenak, Michaela Adamcova, Ludovit Paulis

**Affiliations:** 1 Institute of Pathophysiology, Faculty of Medicine, Comenius University, 81108 Bratislava, Slovakia; jaro.hrenak@gmail.com (J.H.); ludovit.paulis@gmail.com (L.P.); 2 3rd Department of Internal Medicine, Faculty of Medicine, Comenius University, 83305 Bratislava, Slovakia; 3 Institute of Experimental Endocrinology, Biomedical Research Center, Slovak Academy of Sciences, 84505 Bratislava, Slovakia; 4 Department of Cardiovascular Surgery, Inselspital—University Hospital of Bern, Freiburgstrasse 18, 3010 Bern, Switzerland; 5 Department of Physiology, Faculty of Medicine, Charles University, 50003 Hradec Kralove, Czech Republic; adamcova@lfhk.cuni.cz; 6 Institute of Normal and Pathological Physiology, Centre for Experimental Medicine, Slovak Academy of Sciences, 84505 Bratislava, Slovakia

## 1. Introduction

The renin–angiotensin–aldosterone system (RAAS) ranks among the most challenging puzzles in cardiovascular medicine. It participates in the principal homeostatic mechanisms such as regulation of vascular tone, circulating volume, organ perfusion, blood clotting, cardiomyocyte growth and collagen matrix turnover [1,2]. The RAAS is a well-established player in the acute stress reaction, with renin occupying the eminent position. Its crucial role is underscored by a variety of triggering stimuli such as renal hypoperfusion due to hypotension, renal artery narrowing or sympathetic system-induced vasoconstriction or reduced sodium chloride (NaCl) load near the macula densa—all issues triggering renin production via different but interdependent routes. This guarantees that the RAAS is “a friend in need—a friend indeed”. On the other hand, if chronically activated, increased angiotensin II (Ang II) and aldosterone levels in the circulation and tissues result in oxidative overload and chronic inflammation followed by endothelium dysfunction, energy imbalance and fibrocyte proliferation. The results of these processes are the undesirable remodeling of the heart, kidney, brain and vessels. Inappropriate RAAS stimulation is considered to underlie a number of pathologic conditions including hypertension, atherosclerosis complications, heart and kidney failure, mental disturbances and inflammatory damage including acute respiratory distress syndrome (ARDS). Besides Ang II and aldosterone, the RAAS involves a bulk of other biologically active molecules involved in a number of physiological and pathological processes, whose role remains to be elucidated [3,4,5,6,7].

## 2. History of the RAAS: A Continuously Evolving Concept

The story of the RAAS is a thriller in three acts, unfolding over three centuries, without seeming to be coming to an end. At the beginning, the classical substances renin, angiotensin II and aldosterone were described as the principal mechanisms controlling circulating blood volume and blood pressure. In 1898, the physiologists Tigerstedt and Bergman documented that a renal cortex extract, which they called renin, independently exerted a long-lasting pressor effect on the sympathetic system [8]. This principal finding, however, failed to arouse the desired interest until the 1930s, when the Goldblatt group described hypertension development via the clamp-induced constriction of renal arteries [9], which was adopted as “Goldblatt hypertension”. Renin was considered to be an enzyme producing a pressor substance, initially called angiotonin, or hypertensin, and later angiotensin [10]. Ang II-mediated blood pressure control was disclosed to reside in aldosterone-dependent [11] or aldosterone-independent direct sodium-retaining actions in the renal distal tubuli [12]. Furthermore, Ang II was indicated to reduce renal blood flow while simultaneously maintaining the glomerular filtration rate via more pronounced vasoconstriction of the efferent compared to the afferent glomerular arterioles [12]. In the following years, besides hemodynamic regulation, Ang II and aldosterone were shown to exert some phenotypic alterations in terms of parenchymal and interstitial cell growth and proliferation in target organs either via a direct trophic effect or by their hemodynamic actions [13].

A second landmark development occurred in the early 1990s, when Dzau suggested [14] that the RAAS can operate as both an endocrine and autocrine/paracrine (local, tissue) system, while tissue angiotensin concentration may even exceed its plasma levels. It was suggested that the circulating RAAS carries out the short-term hemodynamic effects, while the tissue RAAS exerts, especially, cellular hypertrophy or hyperplasia in a number of target organs, leading to a structural rebuilding of the vascular wall, heart, kidney or brain [14]. More recently, experiments on transgenic animal models have revealed that in most tissues, the RAAS amplifies the actions of circulating Ang II [15]. Although disputes over whether the entire RAAS cascade, including tissue renin production, or just its downstream parts are present in particular tissues have never been settled, the local RAAS was shown to have significant implications for the pathophysiology of cardiovascular alterations [15].

Third, a non-classical pathway opposing the effects of Ang II has been emerging in the past thirty years. The discovery of Ang 1-7 in 1988 [16] started a new era of RAAS investigations, covering the discovery of angiotensin-converting enzyme 2 (ACE2), angiotensin II type 2 (AT2), Mas and Mas-related G-protein coupled receptor D (MrgD) receptors and several novel peptides [17]. The principal finding in 2003 that ACE2 serves as the entrance receptor for the severe acute respiratory syndrome coronavirus (SARS-CoV) [18] launched an exciting race to learn about the relationship between RAAS, coronavirus infections and acute lung injury development. Most recently, with the current SARS-CoV-2 pandemic, the important role of the RAAS in the evolution of the infection and its subsequent complications has re-emerged.

## 3. Therapeutic Interventions with the RAAS

In order for the RAAS to serve as a reliable friend, a dynamic equilibrium must be established. Too much support or help at an inappropriate time can spoil any friendship. In clinical trials, heart failure (HF) patients with the highest level of renin had the worst prognosis. In the early 1990s, Pfeffer and Braunwald and colleagues revealed that blocking the RAAS with the angiotensin-converting enzyme inhibitor (ACEI) captopril not only reduced blood pressure but also attenuated left ventricular remodeling and improved survival in patients with a failing heart after myocardial infarction [19]. Although the 20% mortality reduction was encouraging, it also gave space to address the 80% residual mortality. Angiotensin II type 1 receptor (AT1R) blockers (ARBs) attenuating not the formation but the effect of Ang II aroused new hopes for hindering the effect of non-ACE-mediated tissue Ang II formation and shifting Ang II molecules to interact with the angiotensin II type 2 receptors (AT2Rs) mediating the desirable vasodilative, anti-proliferative and anti-inflammatory actions. However, the results of the ELITE II trial and several other trials failed to fulfill these expectations, exerting an equal end-point benefit compared to ACEIs in HF patients [20]. The supposed additional benefit of ARBs was presumably counterbalanced by the shortage of the potentially desirable effect of decreased bradykinin splitting in the case of ACEI, with downstream beneficial actions of nitric oxide and prostacyclin [20].

For many decades, aldosterone was a Cinderella in the hierarchy of the RAAS, supposedly being the effector molecule participating only in volume regulation via sodium and water reabsorption in the kidney distal tubuli; in addition, ACEI/ARB were believed to also block aldosterone production. The disclosure of aldosterone’s pronounced profibrotic and pro-inflammatory effects in various tissues and the knowledge that, in the chronic course, aldosterone escapes the influence of the RAAS blockade attracted the interest of cardiologists. The aldosterone receptor blocker spironolactone impressively reduced mortality when added to the established therapy in patients with severe heart failure [21]. In line with these data, the idea of pronounced RAAS blocking by the combination of ACEI with ARB or the direct renin inhibitor aliskiren was investigated in a number of prospective trials. However, in the ALTITUDE trial, aliskiren on top of ACEI or ARB in patients with type 2 diabetes and renal impairment increased hard cardiovascular and renal end-points [22], and in the ONTARGET trial, the ACE inhibitor + ARB combination did not show any additional benefit over monotherapy but was associated with more side effects [23]. Since no additional benefit of dual inhibition compared to monotherapy was observed while more side effects occurred, antihypertensive treatment combinations blocking two steps within the RAAS should be avoided [24]. Yet, the situation might be somehow different in patients with heart failure. The CHARM study results on the ACEI + ARB combination and the results of spironolactone addition on top of ACE inhibition in HF patients suggest that dual RAAS inhibition might be an option for a selected group of cardiovascular patients [25].

## 4. COVID-19–RAAS Interactions and Therapeutic Implications

One of the most discussed issues currently is the role of the RAAS in the process of SARS-CoV-2 infection and its complications, including ARDS development. SARS-COV-2, a member of the Coronaviridae family, binds by its spike (S) protein to the cell surface and enters cells via a cell membrane-bound carboxypeptidase angiotensin-converting enzyme 2 (ACE2), expressed in the lungs, heart, vasculature, bowels or kidney [26,27]. The physiological role of ACE2 is to convert Ang I to Ang 1-9 and Ang II to Ang 1-7. Compared to the classical ACE/Ang II/AT1 route, which gives rise to vasoconstriction, hypertrophic growth and fibrosis, coagulation, oxidative stress and inflammation, the ACE2/Ang1-9/Ang1-7/Mas pathway is perceived as a protective way opposing the deleterious action of Ang II by promoting vasodilation and natriuresis, and inhibiting inflammation or fibrosis. This cascade acts via Ang 1-7 binding to the Mas receptor, Ang 1-9 interaction with the AT2 receptor or, when metabolized in an alternative breakdown pathway, via alamandine and MrgD receptor [17,28].

**Figure 1:**
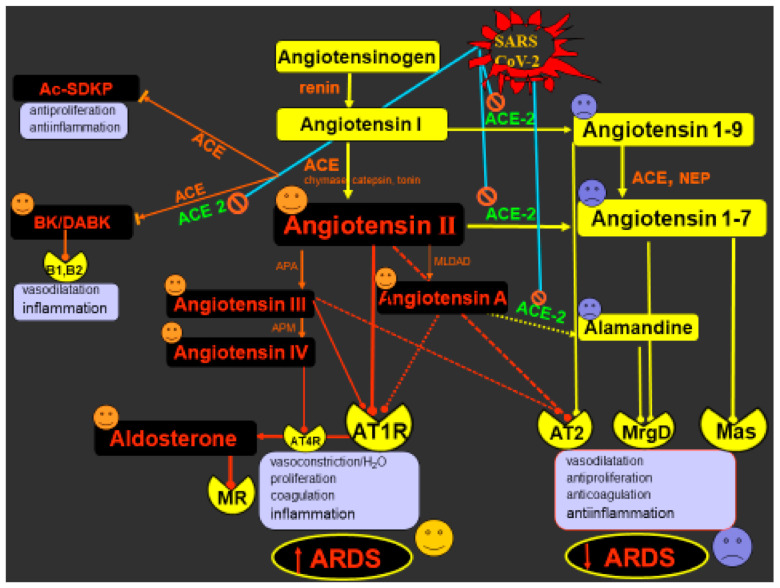
Severe acute respiratory syndrome coronavirus (SARS-CoV-2) interactions with the renin–angiotensin–aldosterone system (RAAS). SARS-CoV-2 binds with membrane ACE2, reducing its membrane expression (by internalization or shedding) and resulting in attenuated formation of angiotensin 1-9, angiotensin 1-7 or alamandine; this results in reduced stimulation of AT2R, Mas and Mrg receptors and their protective cardiovascular and anti-inflammatory actions. On the other hand, downregulated Ang II conversion to Ang 1-7 enhances the bioavailability of Ang II, Ang A and aldosterone, with subsequent stimulation of AT1R and MR resulting in pro-proliferative and pro-inflammatory actions. Both the reduced ACE2/Ang 1-7/Mas/AT2/MrgD protective pathway and the stimulated ACE/Ang II/AT1 deleterious route accelerate inflammation and ARDS development. Furthermore, Ang II is degraded to its active splitting products Ang III and Ang IV by the aminopeptidases A and M. While Ang III can support the action of Ang II via AT1R, Ang IV increases pro-inflammatory genes via interaction with the AT4 receptor. In addition, downregulation of ACE2 may enhance the bioavailability of bradykinin and its proinflammatory effect and the potentially protective N-acetyl-seryl-aspartyl-lysil-proline (Ac-SDKP) is downregulated via ACE. ACE—angiotensin-converting enzyme; ACE2—angiotensin-converting enzyme 2; MR—mineralocorticoid receptor; BK—bradykinin; DABK—des-Arg9-bradykinin; B1, B2—bradykinin receptors; APA—aminopeptidase A; APM—aminopeptidase M; AT1, AT2 and AT4—angiotensin receptor type 1, 2 and 4, respectively; MrgD—Mas-related G-protein coupled receptor D; Mas—Mas receptor, ARDS—acute respiratory distress syndrome, the stimulatory or inhibitory impacts of SARS-CoV2-induced ACE2 inhibition.

SARS-CoV-2 disrupts the fragile balance between the protective and deleterious RAAS pathways. The endocytosis of the SARS-CoV-2/ACE2 complex and the sheddase tumor necrosis factor-α-converting enzyme (ADAM17)-induced cleavage downregulates membrane-bound ACE2 (mACE2) and increases soluble ACE2 (sACE2) [28]. Reduced ACE2 activity results in elevated plasma Ang II levels in COVID-19 patients [29]. In relation to these findings, concerns have emerged regarding the safety of ACEI/ARB usage during the COVID-19 pandemic. In line with experimental data indicating that RAAS inhibitors upregulate the expression of ACE2, concerns were raised that ACEI/ARB could accelerate and aggravate SARS-CoV-2 infection, and therefore, RAAS inhibitors should not be used during the COVID-19 pandemic [30]. On the other hand, a quite opposite view has emerged suggesting that RAAS inhibition might exert protective effects against COVID-19. First, ACEI/ARB reduces the Ang II-mediated pro-inflammatory effects. Second, RAAS inhibition can potentially stimulate the ACE2-mediated formation of Ang 1-7 (from Ang II in the case of ARB) or Ang 1-9 (from Ang I by ACEI) with their anti-inflammatory actions [31]. Third, ACEI treatment increases the levels of N-acetyl-seryl-aspartyl-lysil-proline (Ac-SDKP), which is an alternative substrate for ACE. It is a ubiquitous molecule generated from the N-terminal sequence of thymosin ß4, exerting anti-inflammatory and anti-proliferative effects (Figure 1) [32]. Given the evidence-based mortality and morbidity benefit of ARB/ACEI treatment in cardiovascular pathologies, cardiologic societies have unanimously recommended against ceasing RAAS inhibitor therapy during the COVID-19 pandemic [27,31]. Indeed, a meta-analysis of seven trials with 16,624 COVID-19 patients revealed that ACEI/ARB usage was not associated with heightened sensitivity or with deterioration of prognosis in the population with a COVID-19 diagnosis [33]. Several prospective clinical studies are currently investigating whether RAAS inhibition is safe and even beneficial in SARS-CoV-2 infection [33].

## 5. COVID-19 and the RAAS: Potential Approaches and Perspectives

The uncertainty in predicting the outcomes of RAAS modulation in COVID-19 is caused by the simultaneous modulation of various protective or deleterious RAAS pathways by the virus. Ang II may not only be converted to Ang 1-7 by ACE2 but also to angiotensin A (Ang A), positioned at the cross-road between the deleterious and the protective branches of the RAAS. Ang A can either display a similar action as Ang II via AT1R or be converted to alamandine by ACE2. Alamandine, which can also originate from Ang 1-7 by decarboxylation of its aspartate residue, exerts presumably protective effects such as vasorelaxation, endothelial protection and fibrotic remodeling curbing effects via the MrgD receptor [34]. Similarly, Ang 1-7 itself, arising either from Ang II or from Ang 1-9, could not only act via Mas receptor but also interact with MrgD or AT2R (Ang II type 2 receptor), both of which exert anti-inflammatory, anti-remodeling and hemodynamic protection [2,35]. Of importance, there is a positive feedback interplay between Mas/MrgD and AT2R-mediated routes reinforcing each other; e.g., AT2R stimulation leads to increased Mas and ACE2 expression, and Mas activation supports ACE2 and AT2R expression. This cross-talking of mutual support of protective pathways helps to maintain the balance with the Ang II/AT1R deleterious route [35]. Additionally, both ACE and ACE2 cleave bradykinin, and SARS-CoV-2-induced downregulation of ACE2 or treatment with ACEI can limit bradykinin degradation, increasing its bioavailability and pro-inflammatory effects [36,37]. It is also worth noting that Ang II is degraded to Ang III and IV by aminopeptidase A and M. Both these angiotensins are biologically active. While Ang III enhances the action of Ang II via AT1R, Ang IV activates the NFkB pathway and increases pro-inflammatory genes via the interaction with AT4 receptors [2,38].

Various therapeutic approaches aim to target different levels of the RAAS cascade to attenuate potentially deleterious pathways or stimulate protective ones. However, these interactions are mostly more complex than expected, and the net effect is difficult to predict at the current level of knowledge. Apart from RAAS inhibition, there are several future prospects for RAAS-based therapies in COVID-19. First, Mas receptor stimulation via inhibition of Ang 1-7 degradation or enhancement of its endogenous production by recombinant ACE2 is being studied in clinical trials in COVID-19 patients [35,39,40]. Second, ACE2 is assumed not only to exert anti-inflammatory actions due to Ang II conversion to Ang 1-7; sACE2 maintains its catalytic activity but loses its virus internalization capability, thus serving as a potential decoy for virus particles, preventing their binding to mACE2 and cellular invasion [26,28]. Third, enhancement of the bioavailability of alamandine by ACE2-induced angiotensin A conversion or its external delivery might be another clue for its anti-inflammatory and anti-remodeling effects [32]. Fourth, the dual neprilysin/AT1 inhibitor sacubitril/valsartan increases atrial natriuretic peptide availability with anti-inflammatory action along with the blockade of the toxic Ang II effects, potentially attenuating the threat of an excessive immune response in COVID-19 [41]. Fifth, spironolactone downregulates the TIMPRSS2 protease, which is necessary for spike protein processing by SARS-CoV-2 in the host cell. Spironolactone also inhibits furin, which promotes the virus cell entrance and pulmonary inflammation. The anti-inflammatory effect of spironolactone along with its antifibrotic action should also be considered in the struggle against COVID-19 with pulmonary and cardiovascular damage [42]. Sixth, AT2R agonists, such as compound 21 (C21), could compensate the reduced ACE2/Ang 1-7/Mas signaling and provide compensatory attenuation of the inflammatory storm, endothelial damage and clot formation in the lungs, heart and brain by stimulating AT2 or Mas receptor [35].

Moreover, it seems that the timing of the therapy could be of significant value. In the early phase of pulmonary infection, stimulation of the Ang II pathway recruits inflammatory cells, boosting the defense mechanisms against any infection. However, in the later phase, the relative dominance of the ACE2/Ang 1-7/Mas pathway is desirable to quieten inflammation and prevent excessive damage from the overactivated immune system. If the enhanced activity of the ACE/Ang II/AT1R axis persists in the later phases of infection, the originally defensive process can result in excessive inflammation, cytokinin storm and increased capillary permeability with pulmonary edema manifested as ARDS, possibly followed by fibrotic lung remodeling [6,43]. Thus, it does not seem unreasonable to suppose that the benefit of a therapy modifying the multifaceted RAAS system could be achieved by focusing on a particular period of the SARS-CoV-2 damaging process.

## 6. Conclusions

Taken together, the renin–angiotensin–aldosterone system is a phylogenetically evolved, powerful and well-armed ally for difficult situations. As a sheer friend, it is always ready to fight against every enemy. However, in all wars, both armies suffer. Experience shows that sometimes, more can be achieved by diplomacy than by force. An approach based on a balance between dominance and friendship can help to win not just a particular battle but the entire war.

## Abbreviations

ACE	angiotensin-converting enzyme
ACEI	angiotensin-converting enzyme inhibitor
ACE2	angiotensin-converting enzyme 2
ADAM17	tumor necrosis factor-α-converting enzyme (TACE)
Ac-SDKP	N-acetyl-seryl-aspartyl-lysyl-proline
Ang 1-7	angiotensin-(1-7)
Ang 1-9	angiotensin-(1-9)
Ang II	angiotensin II;
Ang III	angiotensin III
Ang IV	angiotensin IV
APA	aminopeptidase A
APM	aminopeptidase M
ARB	angiotensin II type 1 receptor blocker
ARDS	acute respiratory distress syndrome
AT1R	angiotensin II type 1 receptor
AT2R	angiotensin II type 2 receptor
C21	compound 21
COVID-19	coronavirus disease 2019
DABK	des-Arg9-bradykinin
Mas	receptor Mas
mACE2	membrane-bound angiotensin-converting enzyme 2
sACE2	soluble angiotensin-converting enzyme 2
MrgD	Mas-related G-protein coupled receptor member D
RAAS	renin–angiotensin–aldosterone system
rhACE2	recombinant human angiotensin-converting enzyme 2
SARS-CoV	severe acute respiratory syndrome coronavirus
SARS-CoV-2	severe acute respiratory syndrome coronavirus 2
TNFα	tumor necrosis factor α
TMPRSS2	transmembrane protease serine 2
